# Identification of Geochemical Anomalies Based on RPCA and Multifractal Theory: A Case Study of the Sidaowanzi Area, Chifeng, Inner Mongolia

**DOI:** 10.1021/acsomega.4c02078

**Published:** 2024-05-28

**Authors:** Zhonghai Zhao, Xiang Zhao, Yechang Yin, Chenglu Li, Yuanjiang Yang, Yuping Wang

**Affiliations:** † College of Mining, Liaoning Technical University, Fuxin 123000, Liaoning, P. R. China; ‡ Liaoning Key Laboratory of Green Development of Mineral Resources, LNTU, Fuxin 123000, Liaoning, P. R. China; § Northeast Geological S&T Innovation Center of China Geological Survey, Shenyang 110044, Liaoning, P. R. China; ∥ Heilongjiang Institute of Natural Resources Survey, Harbin 150036, Heilongjiang, P. R. China; ⊥ Liaoning Institute of Geology and Mineral Resources Company, Limited, Shenyang 11000, Liaoning, P. R. China

## Abstract

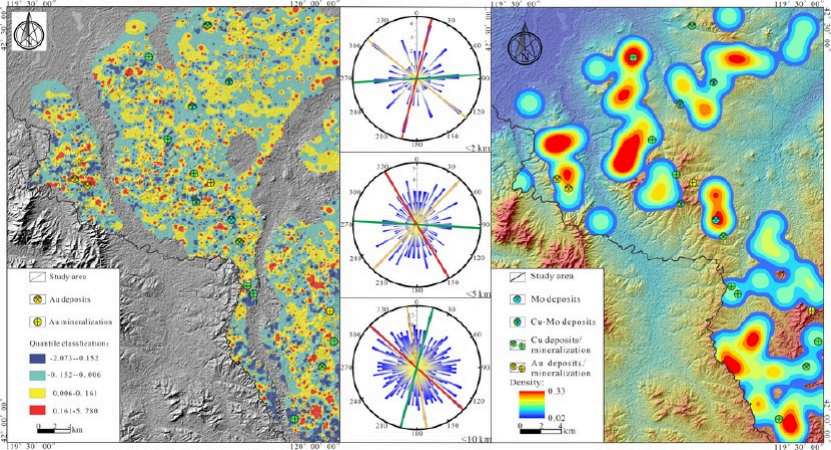

The focus of exploration geochemistry is an accurate interpretation of geochemical data and the precise extraction of anomaly information related to mineralization from complex geological information. However, geochemical data are component data and exhibit a closure effect. Thus, traditional statistical methods cannot adequately reveal and identify the distribution of deep-seated anomaly information. This paper focuses on the Sidaowanzi area in Inner Mongolia and uses multivariate component data analysis methods to process 1:50 000 soil geochemical data. Using the Exploratory Data Analysis (EDA) method, the spatial distribution and internal structure characteristics of raw, logarithmic, and isometric logarithmic ratio (ILR) transformed data were compared and, coupled with robust principal component analysis (RPCA) and elemental component biplots, the association between element combinations and mineralization indicated by these three types of data was revealed. The S-A method was used to decompose composite anomalies of the ILR transformed RPCA score data to extract the characteristics of elemental combination anomalies and background distribution, and the Fry analysis method was utilized to analyze the dominant mineralization direction within the area. The results show that (1) data transformed using the ILR eliminated the influence of the closure effect, making the data more uniform on a spatial scale and exhibiting characteristics of an approximately normal distribution. (2) The S-A method was further used to decompose the composite anomaly of the PC1 and PC2 principal component combinations. The screened-out anomaly and background fields can essentially reflect the ore-causing anomalies dominated by Au and Cu–Mo mineralization. Moreover, the extracted anomalies and background information closely align with known mineral deposits (prospects) and can effectively identify weakly retarded geochemical anomaly information. (3) Fry analysis based on geochemical anomalies indicates that the dominant mineralization directions, by an assemblage dominated by Au and Cu–Mo, predominantly occur in the NE, NW, and proximate EW orientations. The combined application of the aforementioned three methods for the quantitative analysis of geochemical data aims to explore a transferable methodological system, providing new insights and approaches for further prediction of mineralization potential.

## 1 Introduction

Exploration geochemistry is one of the leading methods in mineral exploration and quantitative prediction of mineral resources, widely used for seeking various types of mineral deposits and conducting mineral evaluations.^1−4^ Since the 1970s, geologists have gathered extensive high-quality, multiscale, multielement geochemical data by applying exploration geochemistry techniques. Consequently, how to effectively mine the ore-forming information hidden in geochemical exploration data at different scales and identify such information has become the frontier and a hot topic of study.^5−8^ The related analysis methods mainly include principal component analysis,^9^ factor analysis,^10^ cluster analysis,^11,12^ local enrichment index method,^13^ spatially weighted principal component analysis,^14^ and fractal and multifractal methods.^15−18^ Among them, fractal and multifractal theory’s anomaly identification and extraction method is one of the most straightforward, most effective, fastest, and most reliable methods.

Since Mandelbrot introduced the concept of “fractal geometry” in “The Fractal Geometry of Nature” in 1983, the fractal theory has gradually entered the public view.^19^ It has begun to be applied in analyzing and interpreting various complex phenomena.^20,21^ With continuous research by scholars nationally and internationally, it has been discovered that elemental content exhibits scale invariance and that geological and mineralization processes often create fields characterized by self-similarity or statistical self-similarity, known as multifractal fields.^20,22−24^ On the basis of this theory, various fractal and multifractal data processing models have arisen, including local singularity methods,^25,26^ content-area (C-A),^27−29^ concentration-volume (C-V),^30^ concentration-distance (C-D),^31^ concentration-number (C-N),^32^ spectrum-area (S-A),^33,34^ Fractal projection pursuit classification (FPPC),^35^ spatially weighted singularity mapping (SWSM)^36^ and the number of feature spaces-eigenvalues (N-λ) fractal models.^37^ The aforementioned multifractal methods differ from traditional methods based on frequency distribution. It not only considers the distribution of deep information in geochemical fields but also takes into account spatial correlation, geometric patterns, and scale invariance, allowing complicated to identify effectively can be extraction of weakly retarded geochemical anomalies under complex geological conditions.^4,25,38^ On the basis of the elemental anomaly distribution patterns, the Fry analysis method is introduced and integrated to analyze the characteristics and distribution law of geochemical anomaly deeply, enhance the comprehensiveness of geochemical anomaly information, and establish a transferable method system. This method quantitatively describes the migration and enrichment of elements, thereby determining the dominant mineralization direction of geochemical anomalies. Fry analysis was originally an analytical method used to study the spatial distribution of random points and to evaluate the spatial autocorrelation of rock strain distribution.^39,40^ Previous researchers have built upon this foundation, combining Fry analysis with fractal theory and applying it to various aspects such as mineral distribution analysis, deposit distribution pattern, and mineralization prediction.^41−44^ Currently, Fry analysis, fractal, and multifractal methods are widely used in exploration and geochemical data processing.^15,45,46^

Geochemical data are typically component data with “closure effects”, which can lead to spurious relationship between elements, thus affecting geochemical anomalies’ identification, extraction, and geological interpretation. To resolve these issues, Aitchison and Pawlowsky-Glahn pioneered the establishment of component data analysis theories and methods in the 1980s.^47,48^ Due to influenced by the closure effect, geochemical data belong to Aitchison geometry space, where component data are considered points existing within a simplex space, and decomposed into several mutually independent parts. The specific characteristic of component data is that the sum of all components is a constant value, where each component’s value indicates relative changes rather than absolute values.^49^ To eliminate the influence of the closure effect, building upon prior research on component data, the use of log-ratio transformations for the corresponding spatial conversion to Euclidean space enables more accurate identification and decomposition of geochemical anomalies.^50^ Numerous research examples have shown that the method of log-ratio transformation can more effectively reveal spatial distribution patterns among elements and further accurately identify and decompose elemental background and anomaly information.^11,51^ Presently, widely used log-ratio transformation methods encompass additive log-ratio (ALR), centered log-ratio (CLR), and isometric log-ratio (ILR) transformations, among others.^49,52^

**Figure 1:**
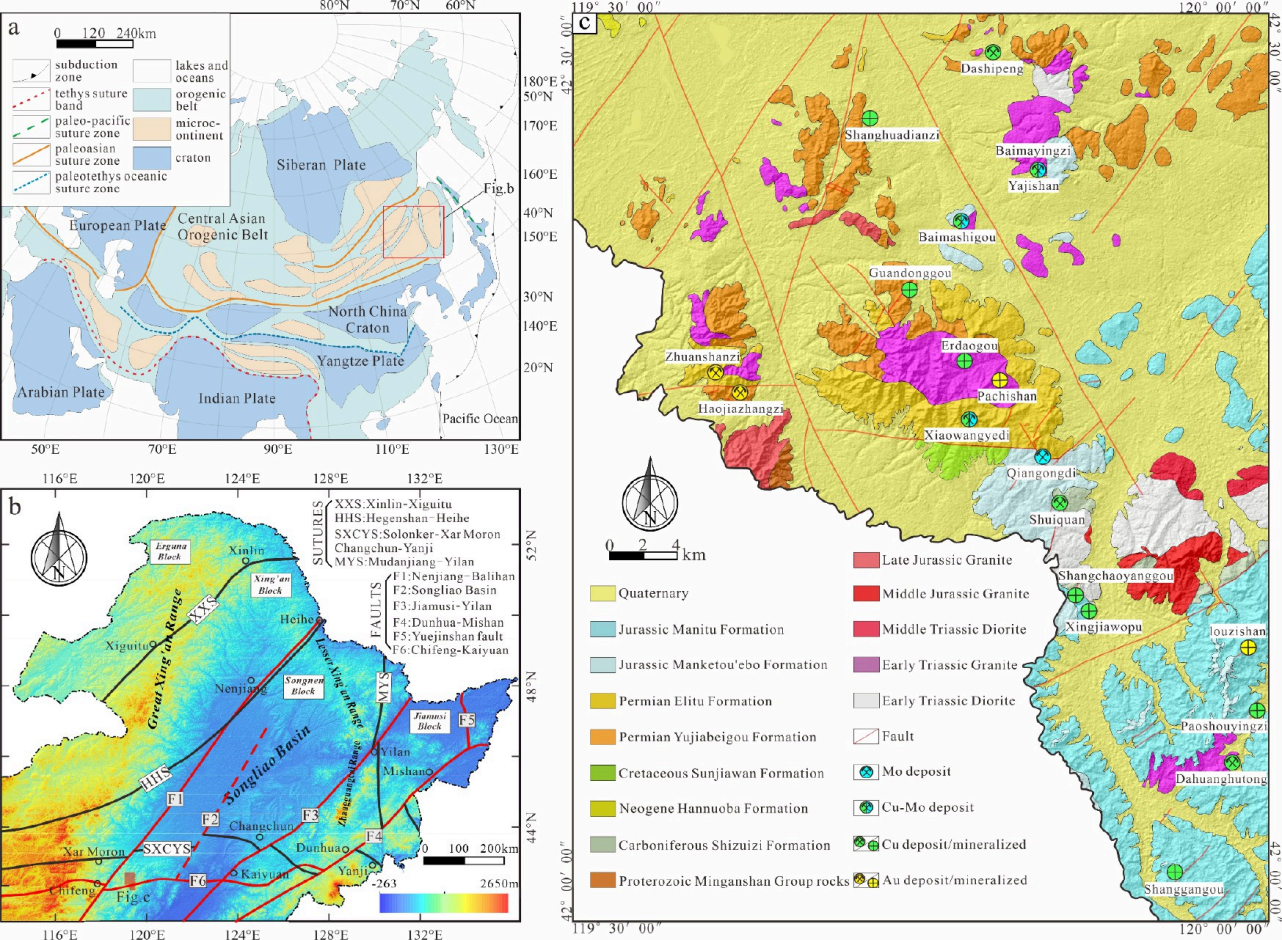
(a) Sketch map of the Central Asian Orogenic Belt and adjacent regions. (b) Tectonic zoning of Inner Mongolia and northeastern China. (c) Geological sketch map of the study area.

With the continuous advancement of mineral exploration, multiple metal ore (deposit) sites (Figure. 1c) have been discovered in the Sidaowanzi area of Inner Mongolia, indicating a high potential for mineralization in the region. However, prolonged mining activities have diminished proven resource reserves and made initiating a new round of exploration in the area an urgent matter. Due to the region’s geological complexity and multiphase nature of mineralization, conventional exploration efforts have been less than ideal. This study employs a combination of multivariate component data analysis methods, fractal theory, and Fry analysis to process nine elements, including Au, Ag, Cu, Pb, Zn, As, Sb, Mo, and Bi, found in the 1:50 000 soil geochemical data of the Sidaowanzi area. Using exploratory data analysis methods, it compares the spatial distribution patterns and structural characteristics of raw data, logarithmic data, and isometric logarithmic ratio transformed data. Subsequently, robust principal component analysis (RPCA) was employed to extract PC1 and PC2 principal component combinations associated with mineralization, and the spectrum-area (S-A) fractal model was used to analyze the spatial distribution pattern of the geochemical data. Finally, by integrating Fry analysis, the dominant mineralization directions in the study area were determined, identifying geochemical anomalies with potential for exploration, thus providing a theoretical basis and guidance for future mining activities in the region.

## 2 Geological Settings

The study area is in the Sidaowanzi region of Inner Mongolia, in the eastern part of the Central Asian Orogenic Belt (Figure 1a).^60^ It is adjacent to the confluence of the northern edge of the North China Craton and the Xing’an Mongolian orogenic belt and belongs to the transitional zone between them (Figure 1b).^53−57,61^ The geological evolution of this area has generally involved various periods, including the collision and amalgamation of the late Paleozoic Amur plate with the North China Craton, Mesozoic intracontinental orogeny the extension and disruption of the North China Craton lithosphere, with the tectonic-magmatic activities of the Mesozoic being particularly intense.^58,59^

The stratigraphic units exposed in the study area are numerous and widely distributed, mainly including Proterozoic, Paleozoic, Mesozoic, and Cenozoic strata. The Proterozoic strata are distributed in the southwestern part of the study area, while the Paleozoic strata are predominantly found in the central and northeastern regions. The Mesozoic strata are situated in the southern part, and the Cenozoic strata are mainly distributed in the central and northern parts, covering a wide area and accounting for more than 38% of the total area. The Proterozoic strata in the study area predominantly comprise the Minganshan Group (Pt_1_*ma*); the Paleozoic strata primarily consist of the Carboniferous Shizuizi Formation (C_1_*s*), the Permian Elitu Formation (P_2_*e*), and the Permian Yujiabeigou Formation (P_2_*y*); the Mesozoic strata chiefly include the Jurassic Manketou’ebo Formation (J_3_*m*), the Jurassic Manitu Formation (J_3_*mn*), and the Cretaceous Sunjiawan Formation (K_2_*sj*). In contrast, the Cenozoic strata primarily comprise Neogene and Quaternary formations (Figure 1c).

The intrusive rocks are widely distributed throughout the region, from the Early Triassic to the Late Jurassic. The lithology is quite complex, ranging from neutral to acidic rocks, with granites and diorites of medium-to-deep formations predominating. The Chifeng-Kaiyuan deep fault extends from the southern part of the study area northeastward and was active multiple times from the Late Jurassic to the Early Cretaceous, resulting in a characteristic dense distribution of mineral deposits (prosepcts) along both sides of the fault.^62,63^ The region is rich in mineral resources and features various types of mineralization, such as epithermal gold, skarn copper, and porphyry copper–molybdenum deposits. Mainly include the Zhuanshanzi gold, Dahuanghutong and Xujiashuiquan copper,^62,63^ Baituyingzi molybdenum,^64^ and Yajishan and Baimashigou copper–molybdenum deposits.^65,66^ Gold deposits represented by Zhanshanzi gold deposit primarily occur in the Permian Yujiabeigou Formation rhyolites and Indosinian granites, exhibiting strong geochemical anomalies of Au and Ag. Copper–molybdenum polymetallic deposits represented by Baimashigou copper–molybdenum deposit are mainly located in high Cu and Mo background areas and are speculated to be related to Mesozoic intrusive rocks and Jurassic strata.^67,68^

## 3 Methods

### 3.1 Data Collection and Analysis

The geochemical data used in this study were obtained from the 1:50 000 soil geochemical survey in the “Inner Mongolia Chifeng City Sidaowanzi Area Mineral Vision Survey Project,” covering an area of 1280 km^2^, in light of regional metallogenic characteristics, screened out nine key elements: Au, Ag, Cu, Pb, Zn, As, Sb, Mo, Bi, and 5185 soil samples were collected. Sample collection strictly adhered to the requirements of the Geochemical Census Specification (1:50 000) (DZ/T0011–2010). The sampling strata predominantly comprised residual and residual slope layers, with the sampling process executed at predetermined locations. Sampling density was established at 1–4 points/km^2^, escalating to 6–8 points/km^2^ at bedrock interfaces and 8 points/km^2^ in focal working zones. Each sample was delivered at a weight of not less than 160 g and sieved with a −4 to +20 mesh. Subsequently, the field-collected samples underwent indoor processing per laboratory specifications, including crushing, drying, and grinding to 200 mesh. The weight of samples meeting the particle size requirements should be equal to or greater than 90% of the weight of the processed samples. The Liaoning Institute of Geology and Mineral Resources analyzed and tested elements in these samples. The quality of the analysis met standard requirements, and the analytical methods and detection limits for the nine elements are detailed in Table 1.

**Table 1:** Analysis Methods and Detection Limit^a^

element	analysis method	detection limit (10^–6^)
Au	CHES	0.0003–0.001
Ag	AAS	0.05
Cu	XRF	2.0
Pb	XRF	5.0
Zn	XRF	10.0
As	AFS	0.5–1.0
Sb	AFS	0.3
Bi	AFS	0.3
Mo	POL	1.0

a AFS, atomic fluorescence spectrometry; XRF, X-ray fluorescence; POL, pscillographic polarography; AAS, atomic absorption spectrophotometry; and CHES, chemi-spectrophotiometric.

### 3.2 Data Processing

#### 3.2.1 Log-Ratio Transformation and Robust Principal Component Analysis

As early as the 1980s, Aitchison and Pawlowsky-Glahn scholars initiated research into the analytical theories and methods for component data.^48,49^ However, geochemical data exhibit a significant characteristic: The total amount of all components equals 1(100%). The components are mutually restrictive and exhibit either negative or positive correlations. This characteristic indicates that elemental correlations are influenced by the closure effect,^49,50^ making it difficult to accurately represent the complex associations between the same variables across different components. Consequently, employing raw data directly for statistical analysis might yield erroneous outcomes.^70^ To more effectively eliminate the closure effect in compositional data, Aitchison (1982, 1986) developed the ALR and CLR transformations,^47,49^ while Egozcues et al. (2003) formulated the ILR transformation.^53^
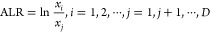
1

As the ALR transformation formula indicates, the lack of uniqueness in the ALR transformation method is apparent, implying that even with the same data set, varying transformation denominators can yield divergent outcomes, thus complicating the analysis and interpretation of the transformed data.
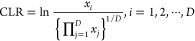
2

To overcome the limitations of the ALR transformation, Aitchison proposed the CLR transform, which utilizes the geometric mean of all variables as the denominator, thereby circumventing the subjectivity inherent in the ALR transformation. Meanwhile, the results of the CLR transformation can be inversely transformed back to the original data set, implying that it also eliminates the asymmetry in the ALR transformation. However, it is evident from the transformation formula that the results of the CLR transformation are singular.
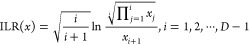
3

Egozcues et al. (2003) proposed the ILR transformation based on the ALR and CLR transformations.^53^ The ILR transformation overcomes the shortcomings of the ALR and CLR transformations in that the distances and angles between the variables in Euclidean space change after the transformation. The ILR transformation ensures that the relative distances between variables remain unchanged before and after the component data transformation. However, the ILR transformation requires selecting a baseline as a reference to establish a coordinate system and display the ratios between components, resulting in one fewer variable post-transformation compared to the original variables.^73^ Furthermore, the ILR transformation also changes the correspondence between variables before and after the transformation, significantly increasing the difficulty of subsequent data analysis and interpretation.^71,72^ To address the interpretational difficulties of the ILR transformation, Filzmoser proposed a method that combines component data from the ILR transformation with robust principal component analysis.^73,74^ This method uses the score and loading values of robust principal component analysis to perform an inverse transformation to the CLR coordinate system under the standard orthogonal basis, thereby establishing a connection with the original data. This approach resolves the complex interpretation problems of the ILR transformation and avoids the singularity issues inherent in the CLR transformation.^52,72^

Additionally, compared to classical principal component analysis, robust principal component analysis is a method developed based on robust statistics. This method employs a robust minimum Covariance estimator as a substitute for the traditional covariance matrix and correlation coefficient matrix, thereby effectively mitigating or controlling the impact of outliers on the outcomes of principal component analysis.^75^

#### 3.2.2 Spectrum-Area Fractal Model

Since the discovery in 1992 that geochemical elemental distributions can be portrayed using power-law relationships, the fractal characterization of geochemical fields has gradually been widely recognized. Various fractal and multifractal models have been generated accordingly, such as the C-A fractal mode,^27−29^ the S-A fractal model,^37,38^ and local singularity analysis methods where the S-A fractal model is a generalization of the C-A fractal model concept. It employs Fourier transformation to convert the elemental geochemical field from the spatial domain to the frequency domain and, based on the anisotropy of elements in the background, anomaly, and interference fields, summarizes their generalized self-similarity. On the basis of this self-similarity, an S-A fractal model filter is constructed. Subsequently, through Fourier inverse transformation, the information filtered by the fractal is reverted from the frequency domain back to the spatial domain, resulting in a decomposed background and anomaly field. The formula for the S-A fractal model is as follows:
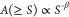
4*S* represents the energy spectral density; β denotes the fractal dimension; *A*(≥*S*) signifies the area exceeding the energy spectral value *S* and ∝ indicates a positive correlation. The energy spectral density *S* and cumulative area *A* are represented in double logarithmic coordinates, and a plot of ln *S*-ln *A*(≥S) is plotted according to the least-squares fitting method (Figure 8), deriving linear and fractal relationships, which decomposing various filters to determine classifier thresholds.^51^

#### 3.2.3 Fry Analysis

Fry (i.e., allocentric distance analysis) was originally an analytical method used to study the autocorrelation of the spatial distribution of rock strain.^78^ This method can reveal the spatial distributions autocorrelation relationships among the target bodies of point elements or near-point elements in space to a certain extent. Subsequently, the method was expanded to study the relative positioning and spatial connections between point elements in space. In recent years, Fry analysis has yielded significant advancements in investigating the spatial distribution patterns of mineral deposits and identifying dominant mineralization trends.^39−41^

The detailed implementation process is illustrated in the accompanying figure, with the critical steps outlined as follows:

(1) Begin with a spatial distribution graph containing n data points as the original image. Please select a specific data point, position it at the center of the graph, and assign a number to it.
(2) Duplicate the original point set and place another point at the center of the diagram, then transfer the data points from the entire duplicated set to the new Fry image.
(3) Repeating step (2) until every point in the original image’s point set has served as a reference for migration in the Fry image, ensuring that the positions of all points are documented to update the Fry image accordingly.

**Figure 2:**
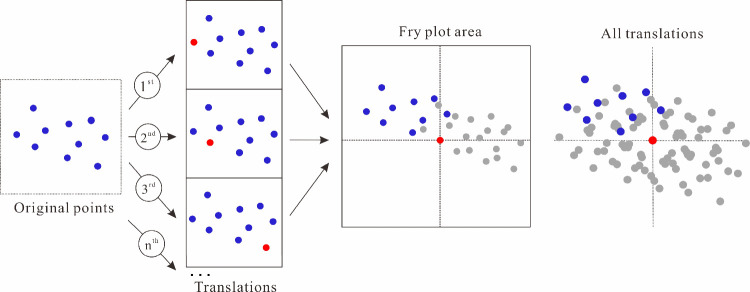
Fry analysis point diagram.

According to the above steps, if the number of known points is *n*, a total of (*n*^2^ – *n*) points can be obtained on the vertical projection diagram, and the projection diagram is called a Fry diagram.^79^ The Fry diagram effectively reveals the overall trend and symmetry of point objects. It can characterize the distance and orientation relationships between the original data points concerning other arbitrary points, thereby increasing the ability to identify the spatial distribution patterns of the original data points. Particularly in situations where there is a lack of data points, or the data points imply very complex spatial distribution patterns, Fry analysis can effectively identify the spatial distribution characteristics of the original data points (Figure 2).

## 4 Results and Discussion

### 4.1 Multivariate Component Data Analysis

**Figure 3:**
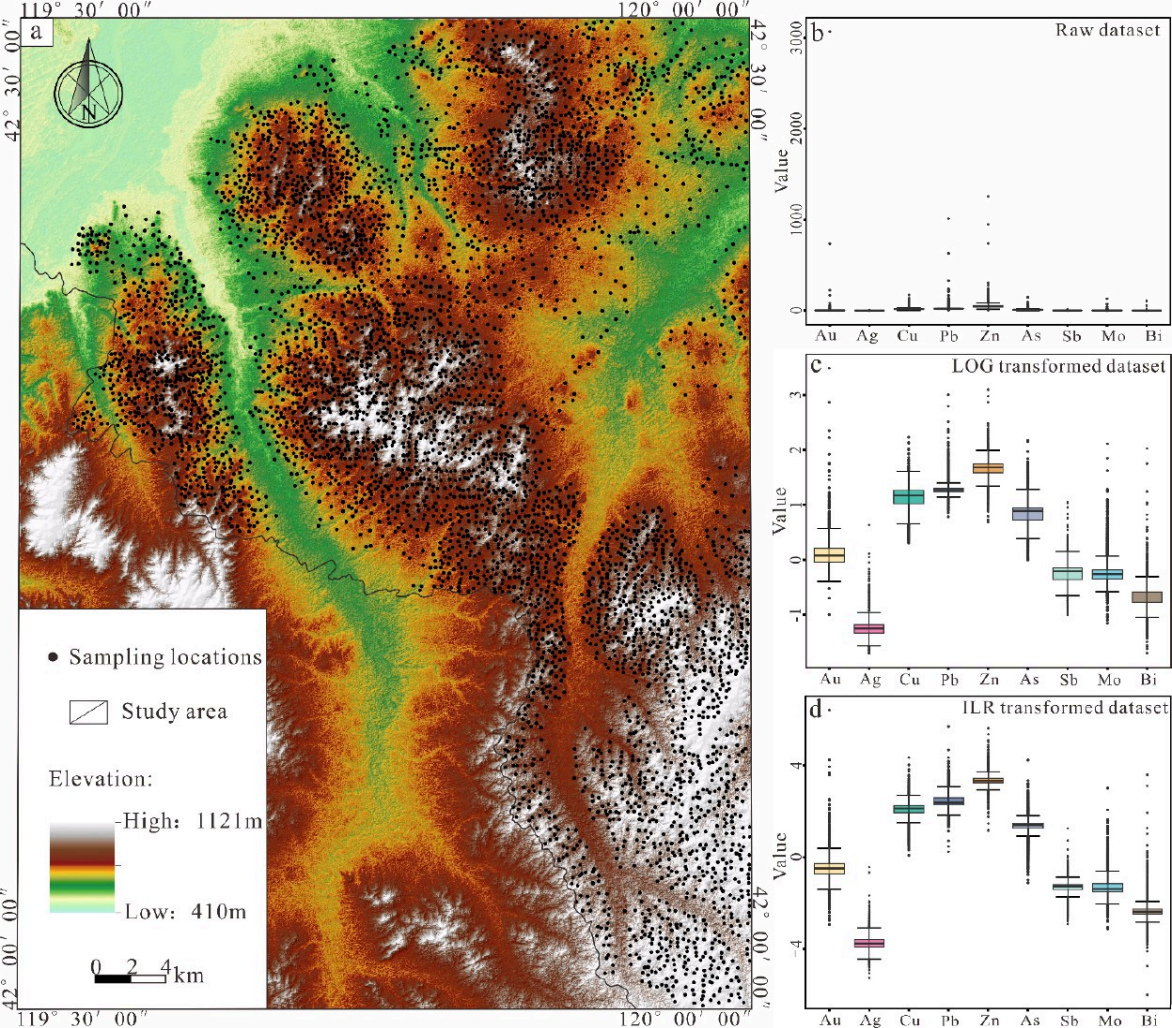
(a) Soil Geochemical Sampling Location Map. (b–d) Box plots of raw data set, Log transformed data sets, and ILR transformed data sets.

The elemental analysis results of 5185 soil samples collected in the study area (Figure 3a) were statistically analyzed. The geochemical characteristics of each element were explored based on the mean of element content, coefficient of variation, and other parameters (Table 2) and statistically analyzed the raw, log-transformed, and ILR-transformed data for the elements. Combined with the exploratory data analysis (EDA) method, it was also used to visualize the data using box plots, density curves, and biplots to obtain the data’s internal structure and distribution characteristics quickly and accurately.

**Table 2:** Statistics of the Raw Data, Logarithmically Transformed Data, and Isometric Log-Ratio Transformation Data of Samples from the Study Area^a^

	element	Au	Ag	Cu	Pb	Zn	As	Sb	Mo	Bi
raw data	maximum	3068.00	4.30	171.00	1012.00	1255.00	147.00	11.10	129.00	106.00
	minmum	0.10	0.02	2.00	6.08	4.92	0.98	0.10	0.07	0.02
	mean	2.23	0.06	14.72	20.26	49.04	7.50	0.60	0.74	0.27
	Cv	19.68	1.18	0.50	0.94	0.63	0.69	0.62	3.08	6.28
	K	3.30	0.88	1.33	0.97	1.04	0.98	1.00	0.81	1.41
	Bv	0.68	69.00	11.10	20.80	47.20	7.65	0.60	0.91	0.19
	25%	0.90	0.07	15.20	20.20	49.30	8.11	0.53	0.96	0.17
percentiles	50%	1.20	0.06	21.30	19.00	60.30	8.25	0.64	0.53	0.26
	75%	1.60	0.08	15.70	18.60	48.90	8.43	0.70	0.57	0.25
	skew	66.15	41.93	5.07	35.52	18.94	10.68	13.73	41.94	53.31
raw	kurt	4555.67	2344.01	76.48	1634.97	619.63	221.25	316.29	2146.70	3092.06
	MAD	0.59	0.01	5.63	2.08	12.75	2.13	0.16	0.15	0.06
	skew	1.46	1.00	–1.02	1.34	0.74	–1.18	–0.59	1.91	2.88
ILR	kurt	16.99	7.79	4.42	6.58	7.47	7.57	7.45	7.02	36.32
	MAD	0.33	0.25	0.20	0.19	0.15	0.15	0.15	0.23	0.17
	skew	1.40	1.25	–0.90	4.30	–0.32	–0.77	–0.54	1.89	1.01
Log10	kurt	14.93	10.65	1.62	39.17	3.98	2.84	2.84	10.33	13.77
	MAD	0.19	0.12	0.16	0.05	0.11	0.12	0.11	0.12	0.13

a Skew, skewness; kurt, kurtosis; MAD, median absolute deviation; Cv, coefficient of variation; K, enrichment factors; Bv, background value. All the element content values are expressed in exponential form but with the exponential part (10^–9^ for Au and 10^–6^ for all the other elements) omitted for the sake of convenience.

As indicated in Table 2, the enrichment factor of the Au element is more significant than 1.5, and its coefficient of variation is 19.68, demonstrating that its distribution is highly uneven and characterized by significant content variations and a high degree of discreteness. It indicates that the migration of the Au element is characterized by enrichment, and the degree of enrichment is high. Additionally, the average value of the Cu element is greater than the background value, indicating relative enrichment characteristics. On this basis, there is a substantial discrepancy between the MAD value of the original data and the mean value of each element, indicating that the various elements within the study area are influenced by various geological factors, leading to considerable disparities in their spatial distribution. Upon comparing three data sets (raw, Log-transformed, and ILR-transformed), the results indicate that the original data exhibit excessively high kurtosis and skewness, presenting a non-normal distribution. In contrast, data subjected to ILR and logarithmic transformations show significant improvements in kurtosis and skewness compared to the original data, aligning with the characteristics of a normal distribution.

**Figure 4:**
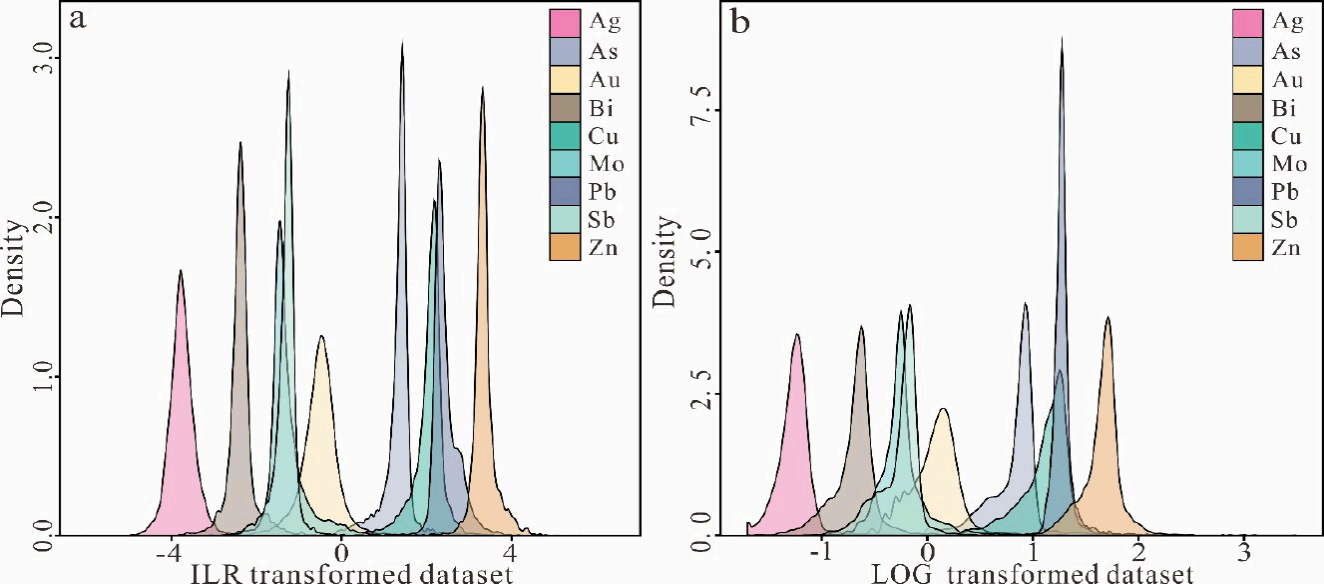
Density curve graphs of different types of data set. (a) Log-transformed data set. (b) ILR transformed-data set.

The raw data in Figure 3b show significant spatial scale differences with a dispersed distribution. High-value anomalies of the Au element result in the suppression of other elements, leading to their distributions not being displayed and presenting a skewed distribution. After the data undergo log transformation and ILR transformation (Figure 3c, d) the spatial spread of the boxplots becomes much more homogeneous, and the differences in the spatial scales of the elements are substantially reduced, bringing the data for each element lie essentially at the same order of magnitude. The corresponding density curve graph (Figure 4) also exhibits an unimodal or multimodal distribution, significantly reducing the variability in the spatial distribution scales of the elements, thereby presenting a normal distribution. However, the original data do not show a corresponding density curve due to the large differences in scale.

Further comparing the boxplot features after logarithmic and ILR transformations, the ILR-transformed data in Figure 3d are more normalized and concentrated in terms of spatial scales and intradata dispersion than the log-transformed data in Figure 3c, which better reflects the trend of the elemental outliers and the high and low extremes distribution of nearly symmetric; the corresponding density curve (Figure 4b) of the ILR-transformed data is closer to the normal distribution than the log-transformed data. So far, the data transformed by the ILR satisfies the characteristics of normal distribution, which tends to be more centered and is more in line with the requirements of multivariate statistical analysis.

In order to better explore the coassociation patterns among the elements in the study area and analyze the impact of closure effects on multivariate component data. This paper performs principal component and robust principal component analysis on the raw, log-transformed, and ILR-transformed data from the study area. Concurrently, the visualization process utilizes biplots, which intuitively display the scores and loadings of the components within the data matrix, thereby illustrating the associations between samples and variables.

**Figure 5:**
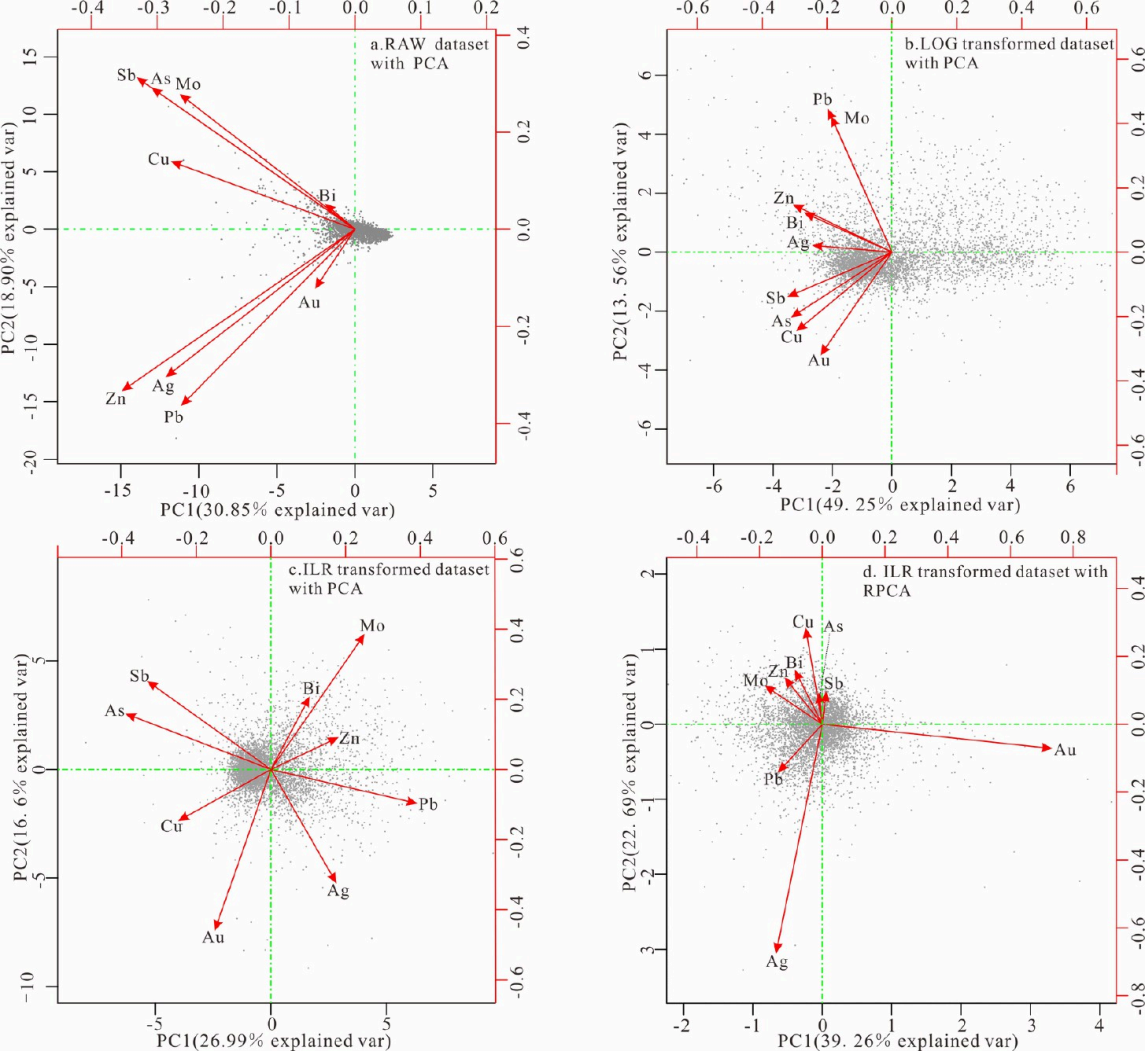
Biplot of PC1 and PC2 principal components for different data sets. (a) Raw data set with PCA. (b) Log-transformed data set with PCA. (c) ILR-transformed data set with PCA. (d) ILR-transformed data set with RPCA.

According to the results of the principal component analysis depicted in the biplots (Figure 5a, b), both the original and the logarithm-transformed data exhibit a nearly identical combinational relationship, characterized by a unilateral trend toward the left, with primary distribution in the second and third quadrants. In the PC1 principal components, all the variables of the above two data have negative loadings, which indicates that the results of the principal component analysis are still constrained and limited by the closure effect and cannot reveal the association between various mineralizing elements. Although there are slight differences in the loading value of elements in the PC2 principal components, the overall distribution pattern remains similar, failing to discern the relationship between element combinations and mineralization. In contrast, following the PCA and RPCA analysis based on ILR-transformed data (Figure 5c, d), it was discovered that the distribution pattern of the elements differs entirely from that of the raw and log-transformed data. All compositional data have been unfolded, with all variables appearing in radioactive patterns, indicating that the closure effect of the data has been eliminated, and the relationships in the transformed data are more distinct. The element combinations presented in the biplots of both methods are relatively similar. However, compared to the PCA method, the RPCA method demonstrates greater robustness to outliers, further reducing the interference caused by data outliers.^75^

Furthermore, the total explained variance by the RPCA method after analysis far exceeds that of the PCA method and is the highest among various component data analysis methods. It can be observed from the biplot sample data that, compared to the raw and log-transformed data, the distribution patterns of various elements in the ILR-transformed data are more uniform, exhibiting an approximately circular uniform distribution in space.

**Figure 6:**
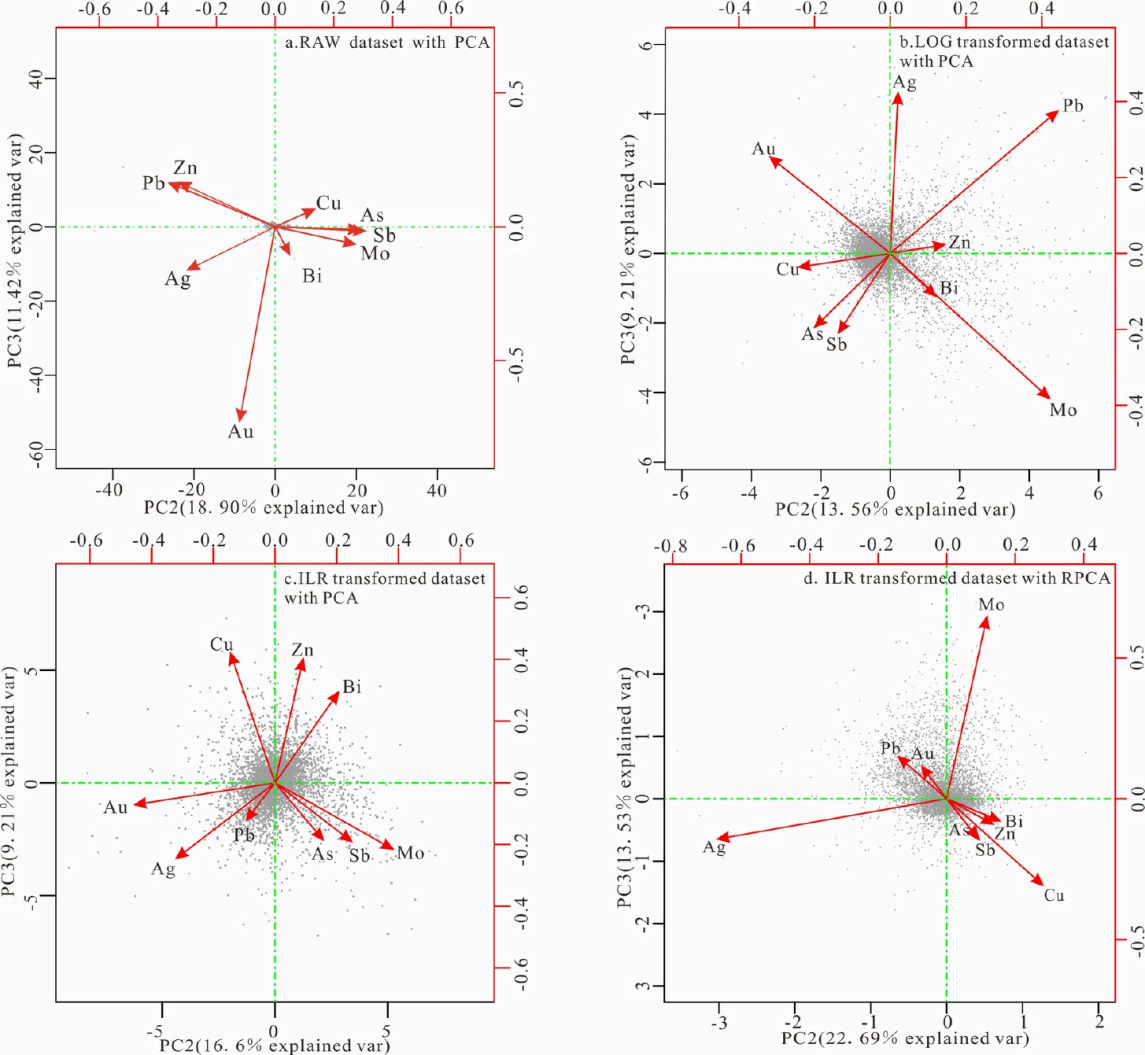
Biplot of PC2 and PC3 principal components for different data sets. (a) Raw data set with PCA. (b) Log-transformed data set with PCA. (c) ILR-transformed data set with PCA. (d) ILR-transformed data set with RPCA.

**Figure 7:**
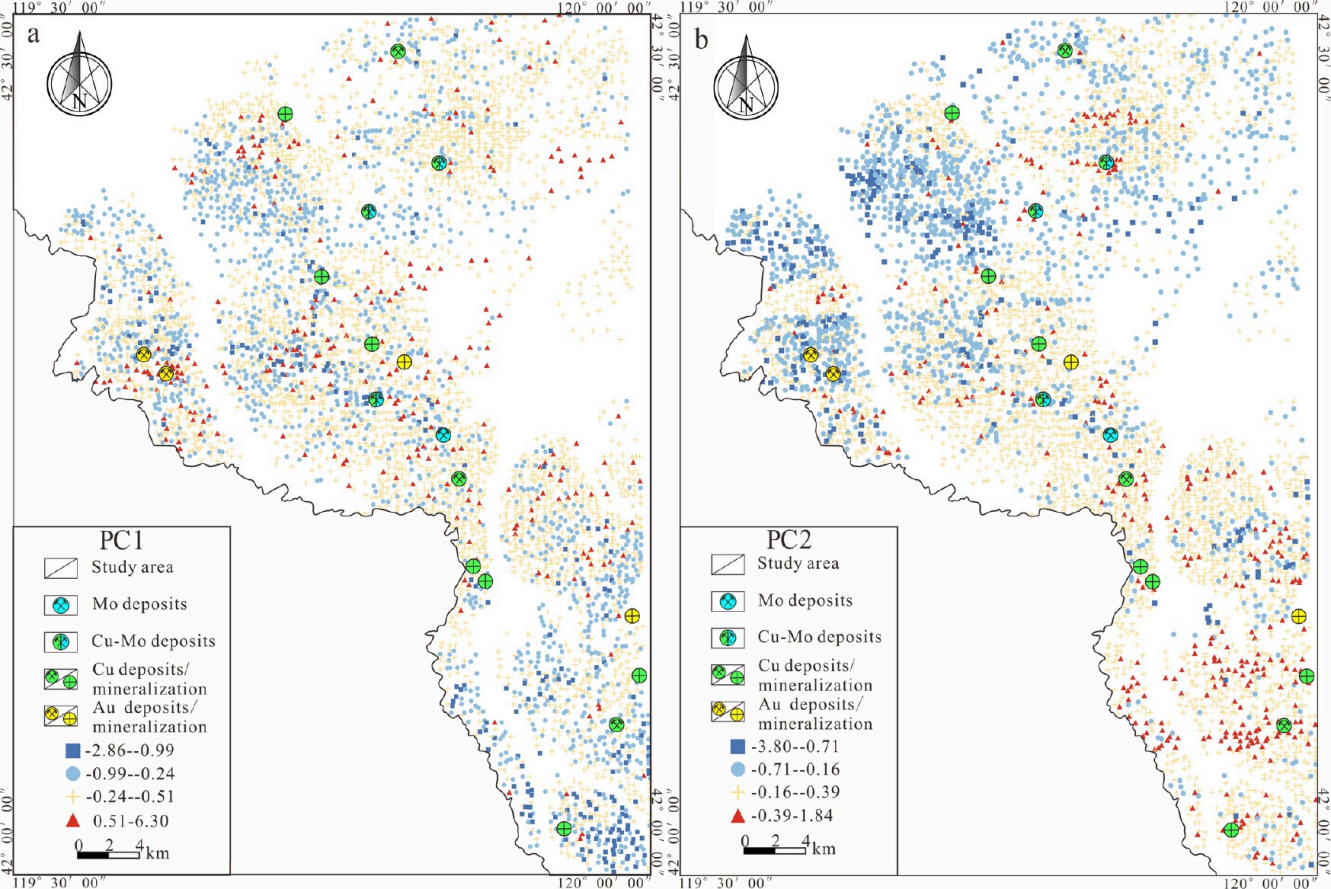
Score and position plot for different principal components. (a) PC1 from RPCA Analysis. (b) PC2 from RPCA Analysis.

According to the biplot of PC2 and PC3 in Figure 6, characteristics similar to those in Figure 5 can be observed. While each element in the biplot of PC2 and PC3 in Figure 6 appears radioactive and seems to have eliminated the closure effect of the component data, the spatial distribution of samples in both the raw and log-transformed data, as observed from the biplots of various types of data, remains uneven, indicating that the closure effect still constrains both. However, the data after ILR transformation, whether in terms of the spatial arrangement of variables or the spatial distribution of samples, show a uniformly dispersed pattern, indicating that the influence of the closure effect has been eliminated. Regarding the biplots (Figure 5), it is important to focus on characteristics such as high-load elements and the angles between elements, which further explain the correlations among elements and their contributions to the principal components.^76,77^ Finally, in the RPCA results after ILR transformation (Figure 5d), it was found that the principal components PC1 and PC2 distinctly identified two sets of element combinations: PC1 with Au–Sb and Ag–Pb–Zn–Cu–Mo–As–Bi, and PC2 with Cu–Mo–Zn–As–Sb–Bi and Au–Ag–Pb. Further combined with Figure 7, it is found that the high-value point of the principal component is consistent with the location of the known gold and copper–molybdenum deposits in the area. It explains that the principal components of PC1 and PC2 reveal the Au–Sb element combination dominated by Au and the Cu–Mo–Zn–As–Sb–Bi element combination dominated by Cu–Mo.

This study used the PC1 and PC2 principal component score data analyzed by ILR transformation and RPCA to draw the principal component score point map (Figure 7). On the basis of the score point map, it was observed that the high values of PC1 and PC2 scores, predominantly Plot of PC1 Scores and Position from RPCA Analysisoccur in the Permian Yujiabigou Formation and Elitu Formation, as well as the Jurassic Manketou’ebo Formation and Manitu Formation within the study area (Figure 1), are the primary ore-hosting strata in the research area. Integrating the geological characteristics of the study area, Figure 7a indicates that the PC1 principal component scores are lower in the southeastern region compared to the northern area. Additionally, the spatial location of the high-scoring area in the west is roughly consistent with the faults’ intersection and alteration zones (Figure 1). The PC2 principal component scores display north–south disparity (Figure 7b), with the high-scoring areas predominantly located in the southeastern part of the study area, chiefly positioned above the Mesozoic Jurassic volcanic-subvolcanic rocks and Early Triassic granite, and have good consistency and spatial correlation with the known Copper and Molybdenum deposits (prospects) in the area.

### 4.2 Spectrum–Area Fractal Model Analysis

**Figure 8:**
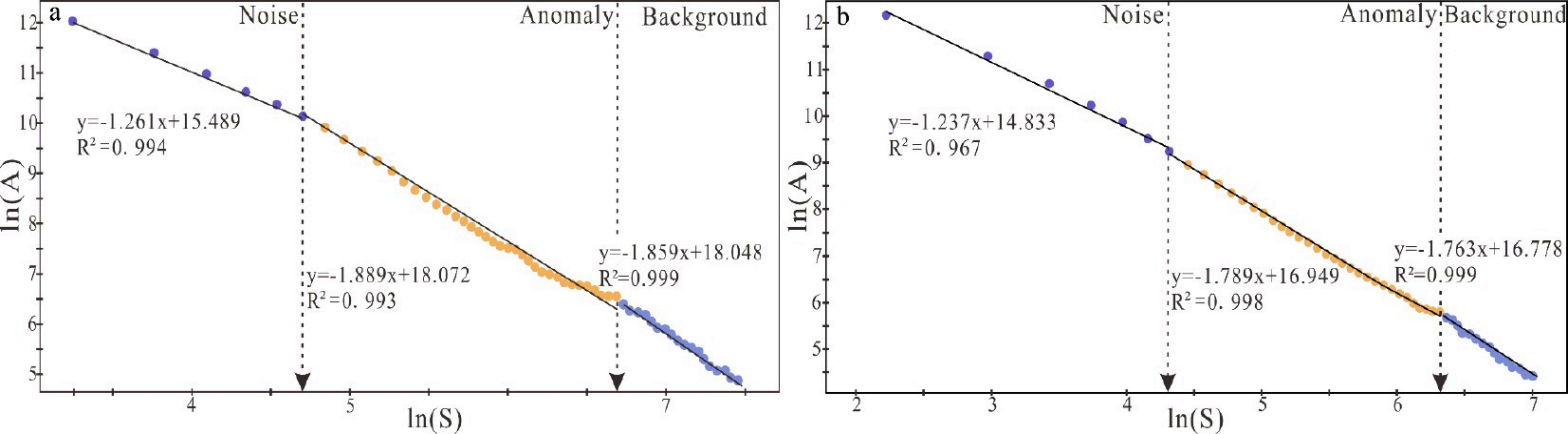
Log–log plot of spectral energy density (S) versus area (A) of PC1 (a) and PC2(b).

To more accurately decode geochemical anomaly information and eliminate the influence of factors such as metallogenesis and regional geology, the RPCA score data based on ILR transformation is further selected for IDW interpolation (inverse distance weighting) and S-A decomposition. This method employs a Fourier transform to translate the score data of the PC1 and PC2 principal components from the frequency domain into the spatial domain, subsequently generating a logarithmic plot of energy spectral density and cumulative area. On the ln *S*–ln *A* (≥S) curve (Figure 8), the least-squares fitting method is employed to ascertain the relationship between the energy spectral density (*S*) and the cumulative area (*A*), with the slope of the fitting curve being indicative of different self-similarity characteristics.^69,70^ In this study, the energy spectral distribution presents lines with different slopes, reflecting that the anomalies distributed possess self-similarity characteristics in the frequency domain that differ from the background, indicating that they belong to the same fractal distribution and are most likely the products of the same process, which further clarification that the S-A method can effectively identify and extract anomalous information.^80−,82^ In the ln *S*-ln *A*(≥S) plot of the PC1 principal component (Figure 8a), the line *y* = −1.261*x* + 15.489 represents the noise field, the line *y* = −1.889*x* + 18.072 represents the anomaly field, and the line *y* = −1.859*x* + 18.048 represents the background field. In the ln *S*-ln *A*(≥S) plot of the PC2 principal component (Figure 8b), the line *y* = −1.237*x* + 14.833 represents the noise field, the line *y* = −1.789*x* + 16.949 represents the anomalous field, and the line *y* = −1.763*x* + 16.778 represents the background field.

**Figure 9:**
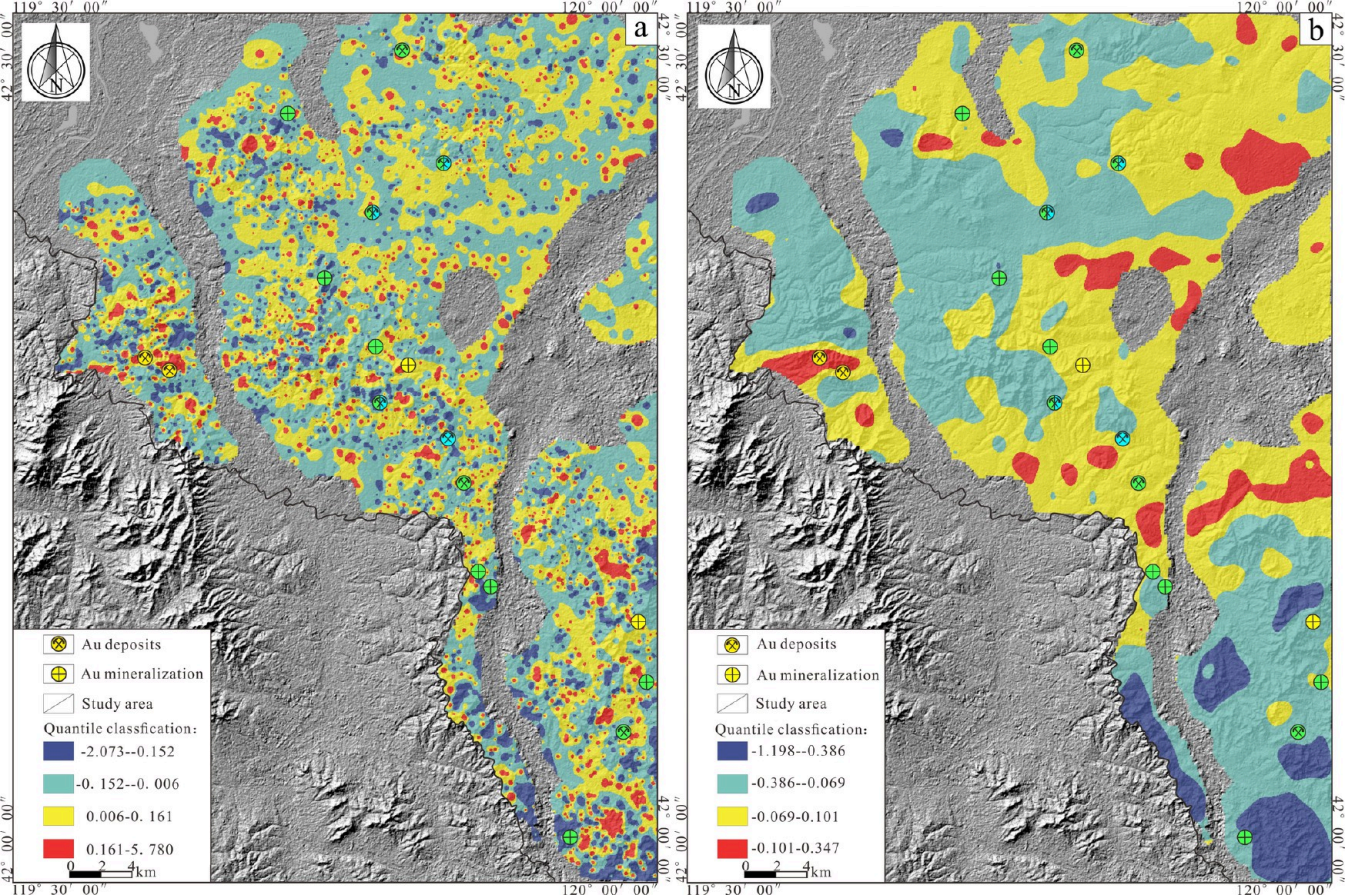
(a) Decomposed anomalous of components PC1. (b) Decomposed background of components PC1.

**Figure 10:**
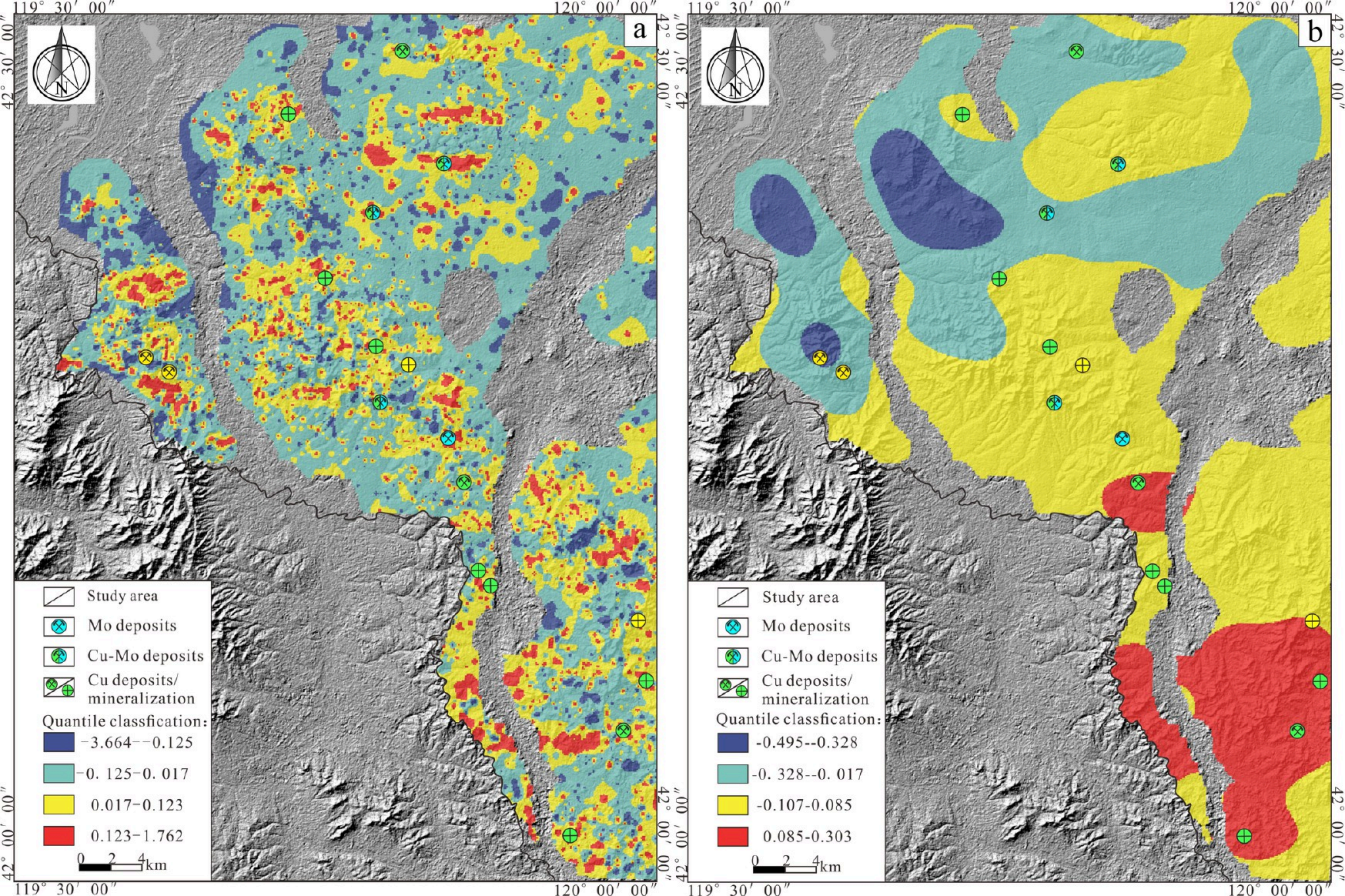
(a) Decomposed anomalous of components PC2. (b) Decomposed background of components PC2.

This study employs the S-A model was used to decompose the composite anomalies of the PC1 and PC2 principal component combination, thereby obtaining the principal component background field and anomaly field in the study area (Figures 9 and 10). Among them, the background field obtained by S-A decomposition mainly reflects the background composition of elemental mass fractions; high-background areas may be favorable for polymetallic mineral exploration; variations in the background strength reflect the presence of elements in a favorable geological context for mineralization, and the anomaly field mainly reflects local anomalous mass molecules of elements and noise generated during data processing.^23,38^

Combining the geological characteristics of the study area (Figure 1) with the decomposed PC1 and PC2 principal component background fields (Figure 9b, 10b), it was found that the spatial positions of high-background areas roughly coincide with the primary ore-hosting strata in the area. Specifically, the high-background regions in the PC1 are predominantly located in the central, northern, and western parts of the study area. In contrast, the areas with low background are primarily concentrated in the northwestern and southeastern parts of the study area. Compared with the anomaly field (Figure 9a), the remaining anomalies obtained after the background discrepancy were removed, not only reducing the extent of the anomalies in the northeast and central part of the study area but also highlighting the weak anomalous information that hidden in the low-background area, and the known gold deposits (prospects) are all located near the high-value area of the anomaly field. Significantly, within the low-background region located in the southeastern part of the study area, prominent high anomalies are observed, predominantly situated in the Upper Jurassic Manketou’ebo Formation and Manitu Formation, and the spatial locations of these anomalies correspond closely with the northeast-trending secondary faults and intrusive rock bodies (Figure 1). Multiple gold polymetallic deposits related to southeastern ore-hosting strata and the volcanic-subvolcanic rocks have been discovered around the perimeter of the study area, suggesting that the high anomaly areas within the low background region in the southeast, possessing significant mineralization potential, are worthy of further exploration.^83,84^

On the decomposed PC2 background field (Figure 10b), it can be seen that the high background areas are mainly located in the southeastern part of the study area and predominantly distributed above the Jurassic strata. Compared with the PC2 anomaly field (Figure 10a), it is found that the known Cu–Mo mineralization points are all distributed in the high-value areas of the anomaly. Comprehensive analysis suggests that the southeastern part of the study area has good mineralization potential, making it a key target for the next stage of mineral exploration in the area.

### 4.3 Fry Analysis Based on Geochemical Anomalies

**Figure 11:**
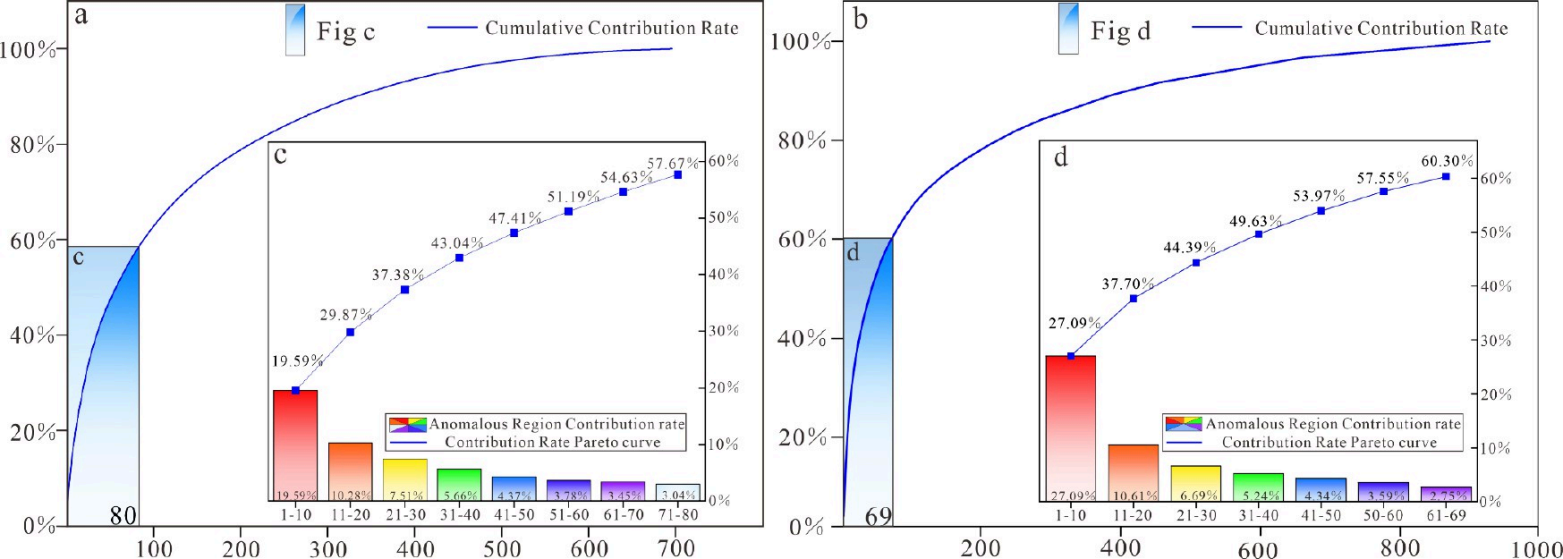
Cumulative area of PC1 (a) and PC2 (b) anomalous area plot. Contribution rate Pareto diagram of PC1 (c) and PC2 (d) anomalous.

In this research, utilizing the element anomaly results ascertained by the S-A fractal model’s background and anomaly fields (Figure 9, 10), a discovery was made that the spatial locations of element enrichment correspond well with the known mineralization deposit (prospects), suggesting that the S-A fractal model, grounded in fractal theory, is effective in identifying anomaly regions predominantly dominated by Au and Cu–Mo mineralization, and in unveiling the potential distribution range of these deposits. On this basis, the spatial location of the elemental enrichment was further utilized as the data basis for the Fry analysis method to rank the anomalous regions identified by the S-A fractal model in terms of area size and calculate the maximum slope change points using the contribution rate graphs (Figure 11a, b), and it was found that the contribution rate change points appeared in the 80^th^ and 69^th^. This is manifested as a gradual decrease in the contribution increase rate, accompanied by a progressive diminishment in the additional contribution to the total variance as the number of areas increases. Therefore, this study selects the 80^th^ and the 69^th^ anomalous regions as representatives, each contributing approximately 60% to the anomaly area, demonstrating strong representativeness.

**Figure 12:**
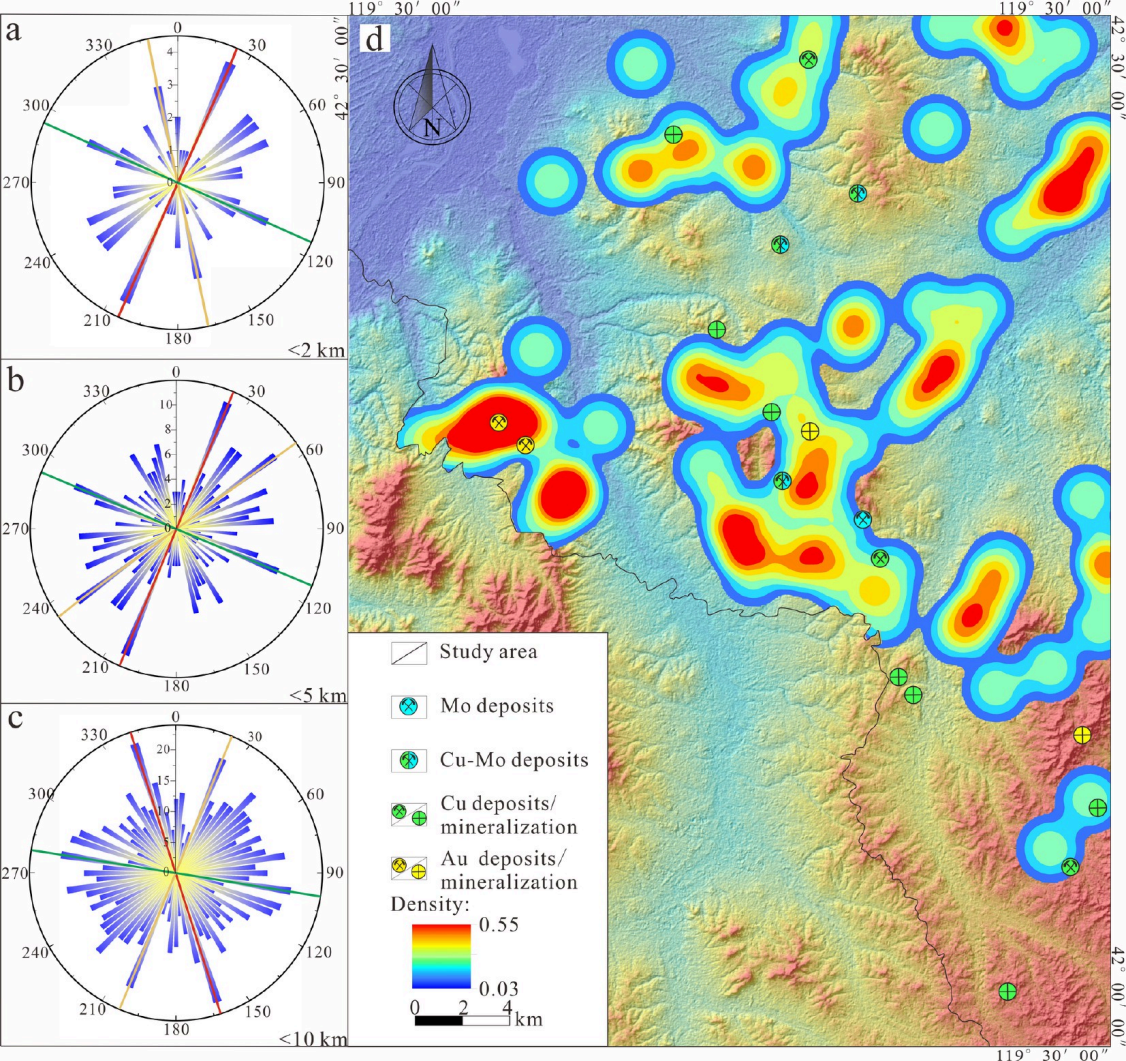
(a–c) Rose diagrams of Au mineralization at different scales. (d) Density map of points analyzed by PC1 Fry.

**Figure 13:**
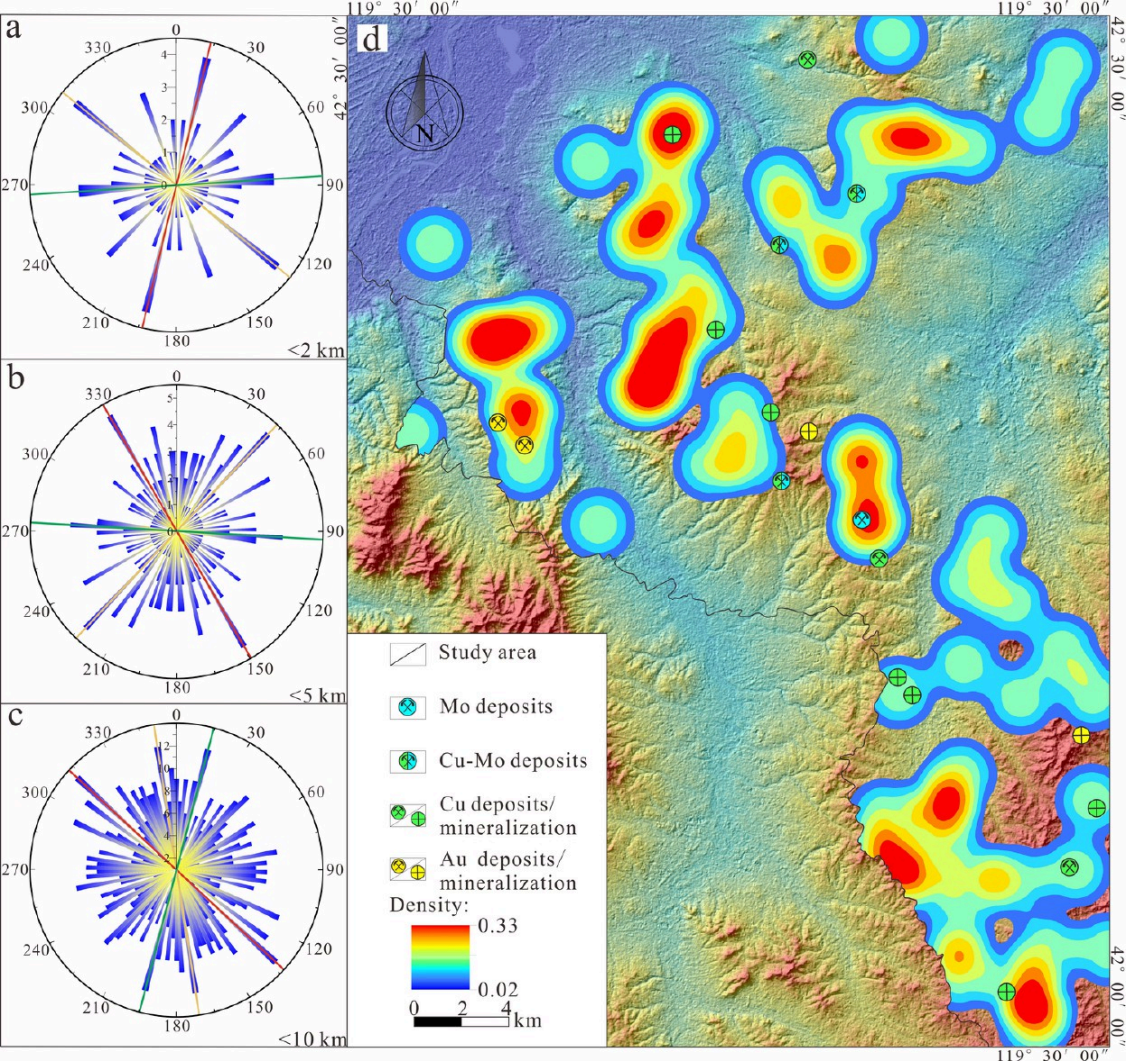
(a–c) Rose diagrams of Cu–Mo mineralization at different scales. (d) Density map of points analyzed by PC2 Fry.

Conducting Fry analysis based on the aforementioned screened anomaly areas (Figure 9, 10), a rose diagram is subsequently created by statistically tallying the number of Fry points in various vector directions from the center point to each point (Figure 12, 13). By analyzing the results of the rose diagrams, the study investigates the spatial distribution patterns of anomalies assemblage dominated by Au and Cu–Mo mineralization at different scales within the research area. The results indicate that within the ranges of 2 km, 5 km, and 10 km, anomalies primarily influenced by Au mineralization exhibit two predominant mineralization directions, NE and NW, along with a secondary, approximately EW direction, while those dominated by Cu–Mo mineralization are primarily concentrated in three dominant mineralization directions, NE, NW, and approximately EW (Table 3).

**Table 3:** Fry Analytical Element Combination and Dominance Mineralization Characterization

fry analysis elemental assemblage	distance (km)	best dominant mineralization direction	suboptimal dominant mineralization direction
Au–Sb	<2	NE(20°–30°)	NW(290°–300°); (340°–350°)
	<5	NE(20°–30°)	NE(50°–60°); NW(290°–300°)
	<10	NW(330°–340°)	NE(20°–30°); approximately EW(280°–290°)
Cu–Mo–Zn–As–Sb–Bi	<2	NE(10°–20°); NW(300°–310°)	approximately EW(260°–270°)
	<5	NE(40°–50°); NW(320°–330°)	approximately EW(270°–280°)
	<10	NW(310°–320°)	NE(10°–20°); NW(340°–350°)

The comprehensive results of the Fry analysis indicate that the predominant mineralization directions in the study area are primarily NE and NW, followed by a secondary approximately EW orientation. Combining the mineral geological characteristics of the study area (Figure 1), the known fault structures within the region, which are relatively well-developed and can be broadly divided into three groups based on their extension directions: NE, NW, and approximately EW, are generally consistent with the predominant mineralization directions identified in this Fry analysis. Furthermore, according to the distribution results of Fry points (Figures 12d and 13d), it is observed that the areas of high density are highly congruent in spatial position and direction with the dominant mineralization directions identified by the Fry analysis, and they are consistent with the geologic setting controlled by structural features. It was also observed that the known mineral deposits (prospects) within the study area are distributed in NE, NW, and approximately EW directions (Figure 1), which to some extent corroborates the reliability of the results obtained from this Fry analysis.

## 5 Conclusions

(1) Data transformed using the ILR method eliminates the influence of the closure effect in the original data, thus more effectively revealing the actual spatial distribution patterns of elements and demonstrating more stable spatial distributions and internal structures. Utilizing the foundation of ILR transformation, the RPCA method was employed to identify elemental combinations associated with mineralization, with the PC1 principal component being an Au–Sb elemental assemblage dominated by Au mineralization and the PC2 principal component consisting of a Cu–Mo–Zn–As–Sb–Bi elemental assemblage dominated by Cu–Mo mineralization.
(2) Employing the S-A fractal model, the geochemical anomaly and background fields of the PC1 and PC2 principal component combinations in the study area were identified and separated. Through the filtered background and anomaly information, mineralization anomalies dominated by Au and Cu–Mo were reflected, which highly corresponded with the known deposits (prospects) within the study area, effectively identifying weakly retarded geochemical anomalies.
(3) Utilizing the Fry analysis method, the spatial distribution patterns of anomalies within the study area were analyzed to characterize their distance and azimuthal relationships quantitatively and to summarize the dominant mineralization directions primarily influenced by Au and Cu–Mo mineralization at different scales, which mainly concentrate in NE, NW, and approximately EW directions. These directions are generally consistent with the significant fault distribution directions within the region, and all known mineral deposits (prospects) within the area are located along these dominant mineralization directions.
